# *In vitro* inhibitory activity of *Bifidobacterium longum* BB536 and *Lactobacillus rhamnosus* HN001 alone or in combination against bacterial and *Candida* reference strains and clinical isolates

**DOI:** 10.1016/j.heliyon.2019.e02891

**Published:** 2019-11-22

**Authors:** Rosanna Inturri, Laura Trovato, Giovanni Li Volti, Salvatore Oliveri, Giovanna Blandino

**Affiliations:** a Department of Biomedical and Biotechnological Sciences, University of Catania, Via Santa Sofia 97, 95123, Catania (CT), Italy; b U.O.C. Laboratorio Analisi II, A.O.U. “Policlinico-Vittorio Emanuele”, Catania, Italy

**Keywords:** Microbiology, Bacteria, Mycology, Microorganism, Peptides, Gastrointestinal system, Bacteriology, *Bifidobacterium longum* BB536, *Lactobacillus rhamnosus* HN001, *Candida* spp., Inhibition growth, Antimicrobial activity, Gastrointestinal pathogens, Urinary pathogens

## Abstract

*Bifidobacterium longum* BB536 and *Lactobacillus rhamnosus* HN001 are two strains frequently used as probiotic components in food supplements. The decrease of potentially pathogenic gastrointestinal microorganisms is one of their claimed mechanisms. The aim of this study was to investigate their ability, alone or in combination, to inhibit *in vitro* the growth of Gram-negative, Gram-positive and *Candida* reference strains and clinical isolates, using different methods.

The cell-free supernatants were obtained by centrifugation and filtration from single or mixed broth cultures and the inhibitory activity was tested using both agar-well diffusion and broth microdilution methods. In order to get some preliminary information about the chemical nature of the active metabolites released in the supernatants, the inhibitory activity was investigated after neutralization, heat and proteolytic treatments.

The highest inhibitory activity was shown by the untreated supernatant obtained from broth culture of the two probiotic strains, especially against bacterial reference strains and clinical isolates. This supernatant showed inhibitory activity towards *Candida* species, too. A decreased inhibitory activity was observed for the supernatants obtained from single cultures and after proteolytic treatment, against bacterial reference strains.

The study suggests that the combination of *B. longum* BB536 and *L. rhamnosus* HN001 could represent a possible alternative against gastrointestinal and urinary pathogens either as prophylaxis or as treatment.

## 1 Introduction

The claimed effects of *Bifidobacterium longum* BB536 and *Lactobacillus rhamnosus* HN001 are: *healthy balance of intestinal bacteria* and *gut health*, respectively [1, 2]. Their claimed mechanism, assuming the general population as their target population, is the decrease of potentially pathogenic gastro-intestinal microorganisms. Numerous *in vitro* and animal studies have been performed to demonstrate their safety [1, 2, 3, 4, 5]. These two strains have been widely used alone as probiotic components in food supplements, thanks to their beneficial effects on human health. Recently, these strains have also been studied in combination to evaluate *in vitro* some of their probiotic features [6].

*B. longum* and *L. rhamnosus* belong to two genera that ferment carbohydrates and produce organic acids (acetic acid, lactic acid, propionic acid), exopolysaccharides and short-chain fatty acids (SCFAs), whose antifungal efficiency is directly proportional to chain length [7, 8, 9, 10]. In particular, bifidobacteria produce acetate and lactate as well as vitamins, antioxidants, polyphenols, and conjugated linoleic acids, whereas lactobacilli produce lactate and small proteins [10, 11, 12, 13]. These bioactive metabolites produced by bifidobacteria and lactobacilli could act as a chemical barrier against pathogen proliferation, contributing to maintaining a correct balance between the microbial populations belonging to the phyla (*Actinobacteria*, *Proteobacteria*, *Bacteroidetes* and *Firmicutes*) normally distributed in the healthy adult human gut [11, 14]. Probiotic bacteria could represent an important strategy to antagonize nosocomial uro- and entero-pathogens, in the era of antibiotic and antimycotic resistance [9, 15, 16, 17, 18, 19, 20, 21, 22, 23].

Our previous studies on *Bifidobacterium longum* BB536 and *Lactobacillus rhamnosus* HN001 investigated their antagonistic activity, when used in combination, and their ability to compete against pathogen adhesion to the HT-29 intestinal cell line. We demonstrated that they do not show antagonistic activity to each other when they are in combination and that they compete with Gram-negatives for adhesion to human intestinal cells [6, 24].

The aim of this study was to investigate the ability of *B. longum* BB536 and *L. rhamnosus* HN001, grown alone or in combination, to produce and release, in the growth medium, metabolites able to inhibit *in vitro* bacterial and *Candida* reference strains and clinical isolates.

## 2 Materials and methods

### 2.1 Strains and culture conditions

The probiotic strains tested in this study were *Bifidobacterium longum* BB536 and *Lactobacillus rhamnosus* HN001. They were provided by Alfasigma S.p.A. (Italy) in March 2017, in individual lyophilized powders. *Bifidobacterium longum* ATCC 15707 and *Lactobacillus rhamnosus* GG were used as control strains. Bifidobacteria and lactobacilli were grown in de Man Rogosa & Sharpe (MRS, Oxoid, Italy) broth or agar supplemented with 0.25% L-cysteine (Sigma Aldrich, Italy - MRSc). Bifidobacteria were incubated for 24–48 h at 37 °C in an anaerobic jar with AnaeroGen sachet 3.5 L (oxygen level: below 1.0%, carbon dioxide level: between 9.0% and 13.0%, Thermo Scientific, Italy); lactobacilli were incubated for 24 h at 37 °C and under aerobic conditions [6].

The reference strains and clinical isolates tested in this study are listed in Table 1. All the reference strains and clinical isolates belonged to the collection of the Bacteriological Laboratory of the Department of Biomedical and Biotechnological Sciences, Section of Microbiology, University of Catania, Italy. Bacterial reference strains and clinical isolates were grown using Brain Heart Infusion (BHI, Oxoid, Italy) broth and Mueller Hinton (MH, Oxoid, Italy) broth and agar, and incubated at 37 °C under aerobic conditions for 18–24 h. *Candida* reference strains and clinical isolates were grown using RPMI-1640 (Roswell Park Memorial Institute medium, Thermo Fisher, Italy) agar and/or broth with the addition of 2.0% w/v of glucose (gRPMI) and Sabouraud Dextrose Agar (SDA, Oxoid, Italy), and incubated at 37 °C under aerobic conditions for 72 h [6, 25, 26].

**Table 1:** Microbial strains tested in the study.

**Reference strains**	
*Escherichia coli*	ATCC 25922
*Klebsiella pneumoniae*	ATCC 700603
*Escherichia coli*	ATCC 35218
*Enterococcus faecalis*	ATCC 29212
*Pseudomonas aeruginosa*	ATCC 27853
*Staphylococcus aureus*	ATCC 29213
*Candida albicans*	ATCC 90028
*Candida krusei*	ATCC 6258
*Candida parapsilosis*	ATCC 22019
**Clinical uropathogenic isolates**	
*Escherichia coli*	061/064
*Escherichia coli*	EC3960
*Klebsiella pneumoniae*	004/027
*Pseudomonas aeruginosa*	018/090
**Clinical enteropathogenic isolates**	
*Escherichia coli*	EC4219
*Salmonella enteritidis*	SEN6
*Salmonella typhi*	STN12
**Clinical isolates from vaginal swabs**	
*Candida albicans*	1-V
*Candida albicans*	2-V
*Candida glabrata*	1-V
*Candida krusei*	1-V
**Clinical isolates from rectal swabs**	
*Candida albicans*	1-R
*Candida albicans*	2-R
*Candida glabrata*	1-R
*Candida krusei*	1-R
*Candida tropicalis*	1-R

### 2.2 Inhibitory assays

The inhibitory activity was tested using supernatants obtained from broth cultures of *Bifidobacterium longum* BB536 and *Lactobacillus rhamnosus* HN001 grown alone or in combination (ratio 1:1), and from broth cultures of control strains grown alone, after 96 h of incubation, using MRSc broth. The cell-free supernatants (CFSs) were obtained by centrifugation (8000 rpm for 15 min) and filtration (0.22μm filter – Millex-GP Syringe Filter Unit, Millipore, Billerica, MA, USA) of the broth cultures [24, 27]. The supernatants were then stored as single-use aliquots at -20 °C until use. The supernatants tested in this study are listed in Table 2.

**Table 2:** Supernatants tested in this study.

Source and features of the supernatant	Name
Supernatant obtained from broth culture of *Bifidobacterium longum* BB536	aBB536-CFS
Supernatant obtained from broth culture of *Bifidobacterium longum* ATCC 15707	aATCC15707-CFS
Supernatant obtained from broth culture of *Lactobacillus rhamnosus* HN001	aHN001-CFS
Supernatant obtained from broth culture of *Lactobacillus rhamnosus* GG	aGG-CFS
Supernatant obtained from broth culture of *Bifidobacterium longum* BB536 and *Lactobacillus rhamnosus* HN001 grown together (ratio 1:1)	aBBHN-CFS
Neutralized supernatant from *Bifidobacterium longum* BB536 to pH = 7.0	nBB536-CFS
Neutralized supernatant from *Lactobacillus rhamnosus* HN001 to pH = 7.0	nHN001-CFS
Heat treated supernatant from *Bifidobacterium longum* BB536	htBB536-CFS
Heat treated supernatant from *Lactobacillus rhamnosus* HN001	htHN001-CFS
Proteinase K treated supernatant from *Bifidobacterium longum* BB536	pkBB536-CFS
Proteinase K treated supernatant from *Lactobacillus rhamnosus* HN001	pkHN001-CFS

### 2.3 Agar diffusion assay

The agar-well diffusion assay was performed modifying the methods described by CLSI M7-A7 for bacteria and CLSI M27-A3 for yeast [28, 29]. Briefly, for assay against bacterial strains, 200 μL of each CFS, listed in Table 2, were dispensed in 6.0 mm wells previously set up in MH agar (Table 1) and for the assay against *Candida* reference strains and clinical isolates, 200 μL of the supernatant aBBHN-CFS were dispensed in 6.0 mm wells previously set up in gRPMI solid agar (Table 1).

Before the assay, bacterial strains were pre-cultured overnight on MH agar and *Candida* strains were pre-cultured on SDA plates. For the inoculum, individual colonies were suspended in 5.0 mL of sterile saline solution (NaCl 0.85% w/v, Sigma Aldrich, Italy) to reach a turbidity corresponding to 1.0 × 10^6−7^ CFU/mL, determined spectrophotometrically (OD_630_ for bacteria and OD_530_ for *Candida*, using a spectrophotometer Bioteck Synergy ht) and 100 μL were spread on agar surface [25, 27, 30]. Sterile MRSc broth was used as a negative control. The inhibitory effect was detected by a zone of inhibition around the well containing the tested supernatant, after 24–72 h of incubation at 37 °C under aerobic conditions. The assays were performed three times in duplicate. The results are expressed as follows: +++ means a very strong inhibitory activity, ++ means a strong inhibitory activity, + means weak inhibitory activity; - means no inhibitory activity.

### 2.4 Broth microdilution assay

The broth microdilution assay was performed inoculating the reference strains and clinical isolates (Table 1) in serial dilutions of tested supernatants, according to the method described by CLSI M7-A7 for bacteria and CLSI M27-A3 for yeast [28, 29].

The supernatants aBB536-CFS, aHN001-CFS, aBBHN-CFS, aATCC15707-CFS and aGG-CFS were tested against bacterial reference strains and the supernatants aBB536-CFS, aHN001-CFS, aBBHN-CFS were tested against bacterial clinical isolates. For the assay, the supernatants were dispensed in 96-well plates (Sigma Aldrich, Italy) and diluted (ranging from 50.0% v/v to 1.5% v/v) in MH broth performing serial two-fold dilutions. The inoculum was prepared suspending individual bacterial colonies, pre-cultured overnight on MH agar, in 5.0 mL of sterile saline solution (NaCl 0.85% w/v). The suspension was adjusted spectrophotometrically to achieve a turbidity of 0.5 McFarland (1.0–2.0 × 10^8^ CFU/mL). The bacterial suspension was then diluted so that, after inoculation, each well of the 96-well plate, contained about 5.0 × 10^5^ CFU/mL [28, 29, 30].

The supernatant aBBHN-CFS was tested against *Candida* reference strains and clinical isolates. For the assay the supernatant was dispensed in 96-well plates and diluted (ranging from 50.0% v/v to 1.5% v/v) in RPMI-1640 medium buffered with MOPS (Sigma, St. Louis, MO, USA), performing serial two-fold dilutions. The inoculum was prepared suspending individual colonies, pre-cultured for 24–48 h on SDA, in 5.0 mL of sterile saline solution (NaCl 0.85%w/v). The *Candida* suspension was adjusted spectrophotometrically (OD_530_) so that, after inoculation, each well of the 96-well plates was 0.5 × 10^3^ to 2.5 × 10^3^ cells/mL, according to CLSI M27-A3 [28, 29, 30].

MH broth and gRPMI (without supernatants) inoculated with the tested strains were used as positive controls, and sterile MH broth and gRPMI were used as negative controls. For bacterial strains, the 96-well plates were incubated at 37 °C for 24 h, according to CLSI M100-S23 [30]; for *Candida* the 96-well plates were incubated at 35 °C for 24–48 h according to CLSI M27-A3 [29].

In order to determine the inhibitory activity of each tested supernatant, the guidelines of CLSI M7-A7 for determining MIC End Points were followed [28]. The lowest concentration of the supernatant that completely inhibited the microbial growth in the wells was detected by eye, compared with the control growth wells (no supernatant added). The microbial growth inhibition was then confirmed by spreading on MH agar or gRPMI agar 100 μL from each well in which the bacterial or *Candida* growth was visibly inhibited, after a spectrophotometric reading of microbial growth (OD_630_ for bacteria and OD_530_ for *Candida*), performed to facilitate reading microdilution tests. The assays were performed three times in duplicate. The results are expressed as follows: +++ means a very strong inhibitory activity, ++ means a strong inhibitory activity, + means weak inhibitory activity; - means no inhibitory activity.

### 2.5 Supernatant inhibitory effects after neutralization, heat and proteinase K treatment

In order to get some preliminary information about the chemical nature of the metabolites released by *Bifidobacterium longum* BB536 and *Lactobacillus rhamnosus* HN001, the supernatants inhibitory activity was investigated towards bacterial reference strains, after neutralization, heat and proteinase K treatment, using the ADM method.

To exclude the effects due to the organic acids, inhibitory activity was tested using supernatants neutralized to pH 7.0 using 0.1 M NaOH (Sigma Aldrich, Italy). To investigate the temperature effects, aliquots of the supernatants were subjected to heat treatment at 121 °C for 15 min [31].

To clarify the inhibitory effects due to the possible presence of peptides, the supernatants were treated with proteinase K (100 μg/mL) at 55 °C for 30 min and then heated (100 °C for 10 min) to inactivate proteinase K [23]. The result was the mean of two individual experiments performed in duplicate.

## 3 Results

### 3.1 Inhibitory activity of the supernatants of *Bifidobacterium longum* BB536 and *Lactobacillus rhamnosus* HN001

The inhibitory activity of the CFSs from broth cultures of *Bifidobacterium longum* BB536 and *Lactobacillus rhamnosus* HN001 grown alone or in combination was evaluated towards different bacterial and *Candida* strains, representative of pathogenic species, using both agar well-diffusion (ADM) and broth microdilution (BDM) methods. The results obtained for untreated acid supernatants against bacterial reference strains are shown in Table 3, the results obtained against clinical bacterial isolates are shown in Table 4 and the results obtained against *Candida* reference strains and clinical isolates are shown in Table 5.

**Table 3:** Inhibitory activity of acid cell-free supernatants from *Bifidobacterium longum* BB536 and *Lactobacillus rhamnosus* HN001 grown alone or in combination against bacterial reference strains using the agar diffusion method (ADM) and the broth dilution method (BDM), in comparison with cell-free supernatants from *Bifidobacterium longum* ATCC 15707 and *Lactobacillus rhamnosus* GG used as control strains.

Bacterial strains	Supernatants#									
	aBB536-CFS		aATCC15707-CFS		aHN001-CFS		aGG-CFS		aBBHN-CFS	
	ADM^a^	BDM^b^	ADM^a^	BDM^b^	ADM^a^	BDM^b^	ADM^a^	BDM^b^	ADM^a^	BDM^b^
*Escherichia coli* ATCC 25922	+	++	++	++	+	++	++	++	+	+++
*Escherichia coli* ATCC 35218	++	++	++	+	+	+++	+	+	++	+++
*Klebsiella pneumoniae* ATCC 700603	+++	+++	++	++	++	++	+	+	+++	+++
*Pseudomonas aeruginosa* ATCC 27853	+	+	++	+	+	+++	+	++	+	+++
*Enterococcus faecalis* ATCC 29212	++	+++	++	++	++	++	+	++	++	+++
*Staphylococcus aureus* ATCC 29213	++	++	++	+	++	+++	++	++	++	+++

# Cell-free supernatants were obtained after 96 h of incubation from broth cultures of *Bifidobacterium longum* BB536 (**aBB536-CFS**)*, Bifidobacterium longum* ATCC15707 (**aATCC15707-CFS**), *Lactobacillus rhamnosus* HN001 (**aHN001-CFS**), and *Lactobacillus rhamnosus* GG (**aGG-CFS**) grown alone and *Lactobacillus rhamnosus* HN001 and *Bifidobacterium longum* BB536 grown in combination (**aBBHN-CFS**).

a For the **agar diffusion method** (ADM) +++ (very strong): diameter of inhibition zone ≥20.0 mm; ++ (strong): diameter of inhibition zone (20.0,15.0] mm; + (weak): diameter of inhibition zone (15.0,10.0] mm; - (no activity): diameter of inhibition zone <10.0 mm; punch diameter =8.0 mm.

b For the **broth dilution method** (BDM) +++ (very strong): ≤12.5%v/v; ++ (strong):25.0%v/v; + (weak):50.0%v/v; - (no activity): >50.0%v/v.

**Table 4:** Inhibitory activity of acid cell-free supernatants from *Bifidobacterium longum* BB536 and *Lactobacillus rhamnosus* HN001 grown alone or in combination against bacterial clinical isolates using the agar diffusion method (ADM) and the broth dilution method (BDM).

Bacterial clinical isolates	Supernatants#					
	aBB536-CFS		aHN001-CFS		aBBHN-CFS	
	ADM^a^	BDM^b^	ADM^a^	BDM^b^	ADM^a^	BDM^b^
*Escherichia coli* EC4219	++	++	++	+++	++	+++
*Escherichia coli* 061/064	++	++	+	++	++	++
*Escherichia coli* EC3960	++	+++	+	++	++	+++
*Klebsiella pneumoniae* 004/027	++	+++	+	++	++	+++
*Pseudomonas aeruginosa* 018/090	++	++	++	+++	++	+++
*Salmonella enteritidis* SEN6	++	++	+	++	++	++
*Salmonella typhi* STN12	++	+++	+	++	++	+++

# Cell-free supernatants were obtained after 96 h of incubation from broth cultures of *Bifidobacterium longum* BB536 (**aBB536-CFS**)*, Lactobacillus rhamnosus* HN001 (**aHN001-CFS**) grown alone or in combination (**aBBHN-CFS**).

a For the **agar diffusion method** (ADM) +++ (very strong): diameter of inhibition zone ≥20.0 mm; ++ (strong): diameter of inhibition zone (20.0, 15.0] mm; + (weak): diameter of inhibition zone (15.0, 10.0] mm; - (no activity): diameter of inhibition zone <10.0 mm; punch diameter = 8.0 mm.

b For the **broth dilution method** (BDM) +++ (very strong): ≤12.5% v/v; ++ (strong): 25.0% v/v; + (weak): 50.0% v/v; - (no activity): >50.0% v/v.

**Table 5:** Inhibitory activity of acid cell-free supernatant from *Bifidobacterium longum* BB536 and *Lactobacillus rhamnosus* HN001 in combination using the agar diffusion method (ADM) and the broth dilution method (BDM).

*Candida* strains	Supernatant aBBHN-CFS#	
	ADM^a^	BDM^b^
***Reference strains***		
*Candida albicans* ATCC 90028	+	++
*Candida krusei* ATCC 6258	+	++
*Candida parapsilosis* ATCC 22019	+	++
***Clinical isolates from vaginal swabs***		
*Candida albicans* 1-V	+	+
*Candida albicans* 2-V	++	++
*Candida glabrata* 1-V	+	+
*Candida krusei* 1-V	+	++
***Clinical isolates from rectal swabs***		
*Candida albicans* 1-R	+	+
*Candida albicans* 2-R	+	+
*Candida glabrata* 1-R	+	+
*Candida krusei* 1-R	+	++
*Candida tropicalis* 1-R	+	+

# Cell-free supernatant was obtained after 96 h of incubation from broth culture of *Bifidobacterium longum* BB536 and *Lactobacillus rhamnosus* HN001 grown in combination (aBBHN-CFS).

a For the **agar diffusion method** (ADM) +++ (very strong): diameter of inhibition zone ≥20.0 mm; ++ (strong): diameter of inhibition zone (20.0, 15.0] mm; + (weak): diameter of inhibition zone (15.0, 10.0] mm; - (no activity): diameter of inhibition zone <10.0 mm; punch diameter = 8.0 mm.

b For the **broth dilution method** (BDM) +++ (very strong): ≤12.5% v/v; ++ (strong): 25.0% v/v; + (weak): 50.0% v/v; - (no activity): >50.0% v/v.

The highest inhibitory capability was shown by the untreated acid (pH = 3.49) supernatant aBBHN-CFS, particularly against bacterial reference and clinical isolates, using the BDM method. The inhibitory effect of aBBHN-CFS towards *Candida* was lower with respect that observed against the bacterial strains. The differences observed between the results obtained from the two different methods could be due to possible chemical-physical interactions between active metabolites present in the supernatant and agar medium. In particular, the supernatant aBBHN-CFS had a very strong inhibitory effect against all tested reference strains; it was active at a concentration ≤12.5% v/v, its effect was higher than those of both aBB536-CFS (pH = 4.31) and aHN001-CFS (pH = 3.47) towards *Escherichia coli* ATCC 25922 (beta-lactamase negative). Moreover, the inhibitory effect of aBBHN-CFS was higher than that observed for aBB536-CFS towards *Escherichia coli* ATCC 35218 (producing TEM-1 beta-lactamase), *Pseudomonas aeruginosa* ATCC 27853, *Staphylococcus aureus* ATCC 29213 (weak beta-lactamase producing strain, *mecA* negative) and higher than that observed for aHN001-CFS towards *Klebsiella pneumoniae* ATCC 700603 (producing SHV-18 Extended-Spectrum Beta-Lactamase, ESBL) and *Enterococcus faecalis* ATCC 29212. The supernatant aBBHN-CFS showed an inhibitory effect higher than that observed for the supernatants aATCC15707-CFS (pH = 3.87) and aGG-CFS (pH = 3.72), used as control, towards all tested reference strains, using BDM (Table 3). When ADM was used, the inhibitory effect of aBBHN-CFS was comparable to that observed for aBB536-CFS (Table 3). Moreover, the supernatant aBBHN-CFS showed a very strong inhibitory activity against the clinical isolates *Escherichia coli* EC4219, *Escherichia coli* EC3960, *Klebsiella pneumoniae* 004/027, *Pseudomonas aeruginosa* 018/090 and *Salmonella typhi* STN12, using the BDM method. A strong inhibitory effect was shown by aBBHN-CFS against *Escherichia coli* 061/064 and *Salmonella enteritidis* SEN6, using the BDM method and all tested bacterial isolates, using the ADM method (Table 4).

The supernatant aBB536-CFS showed a very strong inhibitory effect against *K. pneumoniae* ATCC 700603 using both methods, BDM (25.0% v/v) and ADM (diameter of inhibition zone ≥20.0 mm) and against *E. faecalis* ATCC 29212, using the BDM method (Table 3); against these two strains its activity was higher than those of aATCC15707-CFS, used as control. Moreover, the supernatant aBB536-CFS showed a strong inhibitory effect against *E. coli* ATCC 35218 and *S. aureus* ATCC 29213, using both methods, BDM (25.0% v/v) and ADM (diameter of inhibition zone between 20.0 and 15.0 mm); against *E. coli* ATCC 25922, using the BDM method and against *E. faecalis* ATCC 29212, using the ADM method (Table 3). Tested against clinical isolates, the supernatant aBB536-CFS had a very strong inhibitory effect (≤12.5% v/v) against *E. coli* EC3960, *K. pneumoniae* 004/027 and *Salmonella typhi* STN12, using the BDM method. Moreover, it showed a strong inhibitory effect against *E. coli* EC4219, *E. coli* 061/064, *P. aeruginosa* 018/090 and *S. enteritidis* SEN6, using both methods, BDM (25.0% v/v) and ADM (diameter of inhibition zone between 20.0 and 15.0 mm) and against *E. coli* EC3960, *K. pneumoniae* 004/027 and *S. typhi* STN12, using the ADM method (Table 4). The supernatant aHN001-CFS showed a very strong inhibitory effect against *E. coli* ATCC 35218 and *P. aeruginosa* ATCC 27853 and *S. aureus* ATCC 29213, using the BDM method; a strong inhibitory effect against *K. pneumoniae* ATCC 700603 and against *E. faecalis* ATCC 29212, using both BDM and ADM methods; against *E. coli* ATCC 25922, using the BDM method and *S. aureus* ATCC 29213, using the ADM method. The inhibitory effect of aHN001-CFS against *K. pneumoniae* ATCC 700603 was higher than that of aGG-CFS, used as control (Table 3). Tested against clinical isolates, the supernatant aHN001-CFS showed a very strong inhibitory effect against *E. coli* EC4219 and *P. aeruginosa* 018/090, using the BDM method. Moreover, it showed a strong inhibitory effect against *E. coli* 061/064, *E. coli* EC3960, *K. pneumoniae* 004/027, *S. enteritidis* SEN6 and *S. typhi* STN12, using the BDM method and against *E. coli* EC4219 and *P. aeruginosa* 018/090, using the ADM method (Table 4). The supernatant aBBHN-CFS, which showed the highest inhibitory activity against bacterial strains, was tested against *Candida* (Table 5). It had a strong inhibitory effect (25.0% v/v) against all tested reference *Candida* strains and against the vaginal isolate *Candida krusei* 1-V and the rectal isolate *Candida krusei* 1-R, using the BDM method; and against the vaginal isolate *Candida albicans* 2-V using both methods, BDM (25.0% v/v) and ADM (diameter of inhibition zone between 20.0 and 15.0 mm).

### 3.2 Supernatant inhibitory effects after neutralization, heat and proteinase K treatment

The results obtained for neutralized (pH = 7.0), heat (121 °C, 15 min) and proteinase K treated supernatants tested against bacterial reference strains, using ADM, are shown in Table 6. After treatments, the neutralized supernatants slightly increased their antibacterial activity with respect to the acid ones; the heat-treated supernatants maintained their antibacterial activity comparable with acid ones; and the proteinase K-treated supernatants decreased their antibacterial activity with respect to the acid ones.

**Table 6:** Inhibitory activity of the neutralized, heat and proteinase K treated supernatants from *Bifidobacterium longum* BB536 and *Lactobacillus rhamnosus* HN001 grown alone against indicator strains using the agar diffusion method (ADM).

Bacterial reference strains	Treated supernatants#					
	nBB536-CFS	nHN001-CFS	htBB536-CFS	htHN001-CFS	pkBB536-CFS	pkHN001-CFS
*Escherichia coli* ATCC 25922	++	++	+	+	+	+
*Escherichia coli* ATCC 35218	++	++	++	+	+	+
*Klebsiella pneumoniae* ATCC 700603	++	++	++	++	+	+
*Pseudomonas aeruginosa* ATCC 27853	++	++	++	+	+	+
*Enterococcus faecalis* ATCC 29212	+++	++	++	++	+	+
*Staphylococcus aureus* ATCC 29213	++	++	++	++	+	+

# Neutralized (pH = 7.0) cell-free supernatant from *Bifidobacterium longum* BB536 (**nBB536-CFS**) and *Lactobacillus rhamnosus* HN001 (**nHN001-CFS**); heat treated (121 °C, 15 min) cell-free supernatant from *Bifidobacterium longum* BB536 (**htBB536-CFS**) and *Lactobacillus rhamnosus* HN001 (**htHN001-CFS**); proteinase K treated cell-free supernatant from *Bifidobacterium longum* BB536 (**pkBB536-CFS**) and *Lactobacillus rhamnosus* HN001 (**pkHN001-CFS**). **Agar diffusion method** (ADM) +++ (very strong): diameter of inhibition zone ≥20.0 mm; ++ (strong): diameter of inhibition zone (20.0, 15.0] mm; + (weak): diameter of inhibition zone (15.0, 10.0] mm; - (no activity): diameter of inhibition zone <10.0 mm; punch diameter = 8.0 mm.

The neutralized supernatant nBB536-CFS had a very strong inhibitory effect (diameter of inhibition zone ≥20.0 mm) against *Enterococcus faecalis* ATCC 29212, higher than that observed against this reference strain before neutralization (aBB536-CFS). A strong inhibitory effect (diameter of inhibition zone between 20.0 and 15.0 mm) was shown for nBB536-CFS against all other reference strains (Table 6). Comparing these results with those obtained before neutralization (Table 3), using the agar-well diffusion method, the inhibitory effect generated by nBB536-CFS was lower than that observed for aBB536-CFS towards *Klebsiella pneumoniae* ATCC 700603; comparable to that observed towards *Escherichia coli* ATCC 35218 and *Staphylococcus aureus* ATCC 29213; and slightly higher than that observed towards *Escherichia coli* ATCC 25922, *Pseudomonas aeruginosa* ATCC 27853 and *E. faecalis* ATCC 29212. The neutralized supernatant nHN001-CFS had a strong inhibitory effect against all the tested reference strains (Table 6). Comparing these results with those obtained before neutralization (Table 3), using the ADM method, the inhibitory effect generated by nHN001-CFS was comparable to that observed for aHN001-CFS towards *K. pneumoniae* ATCC 700603, *E. faecalis* ATCC 29212 and *S. aureus* ATCC 29213 and slightly higher than that observed towards *E. coli* ATCC 25922, *E. coli* ATCC 35218, and *P. aeruginosa* ATCC 27853.

The heat treated supernatant htBB536-CFS showed a strong inhibitory effect against all reference strains except *E. coli* ATCC 25922, comparable with that observed before treatment (aBB536-CFS) against all reference strains except for *K. pneumoniae* ATCC 700603, towards which a lower inhibitory effect was observed, and for *P. aeruginosa* ATCC 27853, towards which a slightly higher inhibitory effect was observed (Table 3). The heat treated supernatant htHN001-CFS showed a strong inhibitory effect against *K. pneumoniae* ATCC 700603, *E. faecalis* ATCC 29212 and *S. aureus* ATCC 29213 (Table 6), the results were comparable with those observed for aHN001-CFS against all reference strains (Table 3).

After proteolytic treatment, the supernatants pkBB536-CFS and pkHN001-CFS had a weak inhibitory activity (diameter of inhibition zone between 15.0 and 10.0 mm) against all tested bacterial reference strains. In particular, the inhibitory effect shown by pkBB536-CFS was comparable with that observed before treatment (Table 3) towards *E. coli* ATCC 25922 and *P. aeruginosa* ATCC 27853. The inhibitory effect of pkHN001-CFS was comparable with that observed before treatment (Table 3) towards *E. coli* ATCC 35218, *E. coli* ATCC 25922 and *P. aeruginosa* ATCC 27853.

## 4 Discussion

According to a recent overview, gastrointestinal infections, especially diarrheal diseases, are one of the major causes of morbidity and mortality worldwide [32]. Although the antibiotic treatment has significantly improved health, their overuse is associated with the development and dissemination of specific resistance mechanisms, contributing to the emergency of antimicrobial resistance due to which over 700,000 patients die globally every year [32, 33]. Imbalance between the microbial populations belonging to the main phyla distributed in the adult human gut has been documented in patients with gastrointestinal and urinary infections [11, 16, 17, 18, 19, 20, 21, 22, 23, 34]. Several studies have demonstrated that bifidobacteria and Lactic Acid Bacteria (LAB) are able to competitively exclude pathogenic bacteria and yeasts, either directly, through interactions with pathogenic strains, or indirectly, through the production of active metabolites and the induction of host immune defense [33, 35]. Probiotics could, therefore, represent a potential alternative to conventional antimicrobials either as prophylaxis or as treatment of gastrointestinal infections and for these reasons they remain one of the main means to contrast these infections [33, 36]. The strains, currently used as probiotics, belonging to genus *Bifidobacterium* and *Lactobacillus,* which are normally present in the human intestinal microbiota and are able to produce antimicrobial metabolites such as organic acids, hydrogen peroxide, ethanol, diacetyl, acetaldehyde, saturated or unsaturated free fatty acids and other compounds such as peptides and bacteriocins [22, 37, 38]. These ribosomally synthetized peptides are often active also against drug-resistant pathogens of clinical importance with several mechanisms of action causing different cell membrane damage [33]. Hence, from a probiotic research concept, several studies have demonstrated the antimicrobial activity of supernatant obtained from broth cultures of probiotic strains but few studies have reported effects due to supernatants obtained from co-cultured probiotic strains. In the current study the antimicrobial activity of the supernatants, obtained from broth cultures of the probiotic strains *Bifidobacterium longum* BB536 and *Lactobacillus rhamnosus* HN001 grown alone and in combination, was investigated *in vitro* against Gram-negative, Gram-positive and *Candida* reference strains and clinical isolates, using both agar-well diffusion and broth microdilution methods. Both agar-well diffusion and broth microdilution methods showed comparable results relative to the inhibitory activity of the specific tested supernatant, although a slightly higher activity was obtained using the broth microdilution method. These differences could be due to interactions between agar meshes and antimicrobial substances with a hydrophobic nature presents in the supernatants [16, 17, 18, 19, 20, 21, 22, 23, 27, 34, 37]. The highest inhibitory activity was observed for aBBHN-CFS, which had the lowest pH and was active against all tested bacterial and *Candida* reference strains and clinical isolates. These results confirmed those reported in the literature for supernatants obtained from mixed cultures of different strains of *Lactobacillus* spp. and *Bifidobacterium* spp., it seems, in fact, that strains in co-cultures may produce short chain fatty acid and other active metabolites in varying proportions, showing a synergic effect [39]. Moreover, the results confirmed the effects of the strains *B. longum* BB536 and *L. rhamnosus* HN001 used in combination to act against the bacterial clinical isolates in order to impede the adhesion to the HT-29 human intestinal cell line; other studies present in the literature demonstrated that the production of antimicrobial compounds by probiotic strains contribute to inhibit the adhesion of pathogenic bacteria [6, 35, 39]. The very strong inhibitory activity observed for the acid supernatants aBB536-CFS and aHN001-CFS against some reference strains and clinical isolates could be due to the combination of the effect of different metabolites such as lactic acid, acetic acid, small peptides and bacteriocins released in the supernatant by the producing strains. Different studies have suggested the productions of these active metabolites by the tested probiotic strains [27, 34, 38].

The supernatant aBBHN-CFS, which had showed the highest inhibitory activity against bacterial strains, was also tested against different *Candida* species showing an inhibitory activity from strong to weak. Studies reported in the literature supported these results, demonstrating that strains of *Lactobacillus* spp. and *Bifidobacterium* spp. are able to produce metabolites such as organic acids, H_2_O_2_ and bacteriocin-like substances, which may interfere with growth, morphogenesis, hyphal formation and adhesion of *Candida* spp. [20, 40, 41, 42, 43].

Compared with the acid supernatants, after neutralization, the supernatants nBB536-CFS and nHN001-CFS showed an increased or comparable inhibitory activity against reference strains with the exception of *Klebsiella pneumoniae* ATCC 700603 against which the supernatant nBB536-CFS was less active. The decrease of inhibitory activity of the supernatants pkBB536-CFS and pkHN001-CFS, treated with proteinase, against bacterial reference strains, with respect to the supernatant aBB536-CFS and aHN001-CFS, could be due to the inactivation of the produced bacteriocins or small peptides which have their maximum activity at acidic pH (from 2.0 to 5.0) and could be resistant to heat treatment, as reported in literature [31, 34, 37, 44, 45, 46].

In conclusion, the antimicrobial activity of *B. longum* BB536 and *L. rhamnosus* HN001, alone or in combination, might be due to their production of different metabolites with antimicrobial activity in addition to organic acids. The metabolites released in the supernatants are heat stable. The results using *B. longum* BB536 and *L. rhamnosus* HN001 grown in combination seem to be promising, with respect to each strain grown alone. Further studies are necessary to better characterize possible clinical applications, especially against gastrointestinal and urogenital pathogens.

## Declarations

### Author contribution statement

Rosanna Inturri: Performed the experiments; Wrote the paper.

Laura Trovato: Performed the experiments.

Giovanni Li Volti: Contributed reagents, materials, analysis tools or data.

Salvatore Oliveri: Analyzed and interpreted the data.

Giovanna Blandino: Conceived and designed the experiments; Analyzed and interpreted the data.

### Funding statement

The work was supported by a grant from University of Catania FIR 2016-18 and an unrestricted grant from Alfasigma SpA.

### Competing interest statement

The authors declare no conflict of interest.

### Additional information

No additional information is available for this paper.
